# Volumizing thread lift for subzygomatic arch (lateral cheek) depression

**DOI:** 10.1111/srt.13794

**Published:** 2024-06-17

**Authors:** Jovian Wan, Soo‐Bin Kim, Lisa Kwin Wah Chan, Kar Wai Alvin Lee, Hugues Cartier, Kyu‐Ho Yi

**Affiliations:** ^1^ Asia‐Pacific Aesthetic Academy Hong Kong Hong Kong; ^2^ Division in Anatomy and Developmental Biology Department of Oral Biology Human Identification Research Institute, BK21 FOUR Project Yonsei University College of Dentistry Seoul South Korea; ^3^ EverKeen Medical Centre Hong Kong Hong Kong; ^4^ Centre Médical Saint Jean Arras France; ^5^ Maylin Clinic (Apgujeong) Seoul South Korea

Dear Editor,

Subzygomatic arch depression frequently manifests in elderly individuals as a result of masseter muscle and fat atrophy.^1^, ^2^ Nonetheless, this depression may also appear prominent in younger individuals with well‐defined zygomatic arches. Literature on managing subzygomatic arch depression is sparse, leaving a gap in understanding optimal treatment approaches. Yi et al.^3^ conducted a cadaveric study investigating hyaluronic acid (HA) filler injection for correcting subzygomatic arch depression and enhancing facial contours. While HA filler injection is a popular and minimally invasive method for such corrections, the conventional single‐layer injection technique has limitations, including inadequate volume addition and potential for undesired undulations and spreading. The study provides anatomical guidance, suggesting the use of both hard‐type and soft‐type HA fillers injected into the sub‐superficial musculo‐aponeurotic system (SMAS) and subcutaneous fatty layers, respectively, to maximize correction. Notably, the subzygomatic region's robust and resilient retaining ligaments present challenges for achieving volumization solely through filler administration. Therefore, incorporating volumizing thread lifts into the procedure could offer significant advantages.^4^, ^5^, ^6^

Our aim is to present a successful case of managing subzygomatic arch depression using volumizing thread lifts and to demonstrate the thread lift insertion technique (Figure 1) through a Video S1.

**FIGURE 1:**
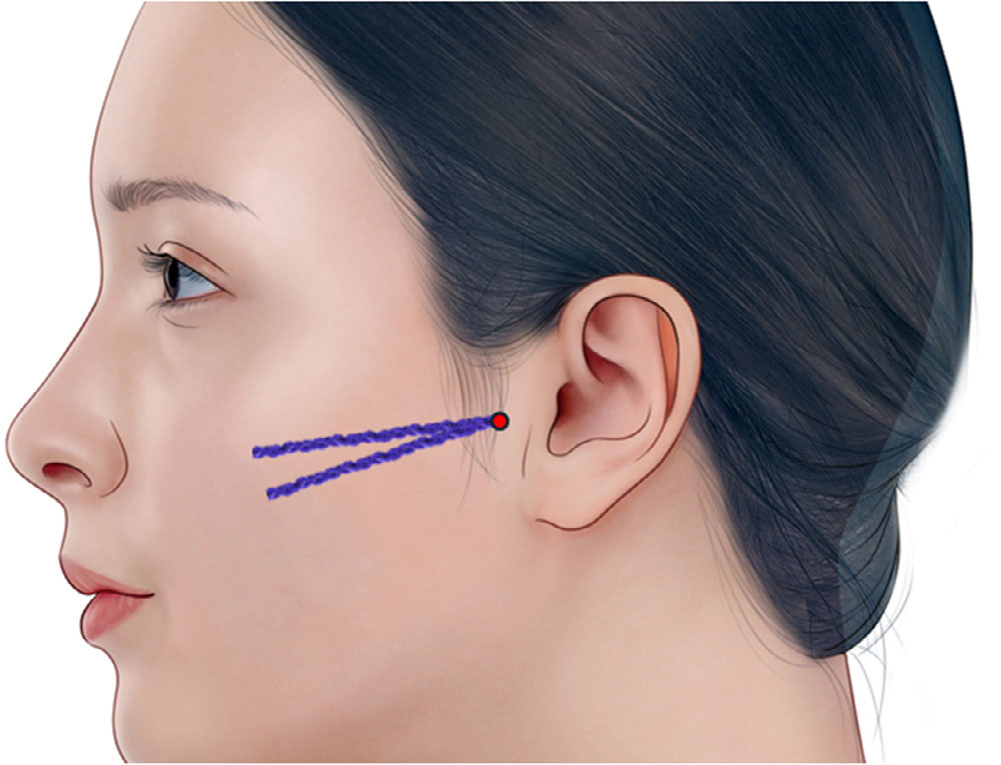
To address subzygomatic arch depression, a correction was performed using an 18‐gauge, 5 cm N‐scaffold (N‐finders, Korea). For subzygomatic arch depression, thicker and firmer volume threads are typically employed, and these can be volumized using N‐Scaffold (N‐Finders, Korea).

A 45‐year‐old patient presented with hollowing under the cheekbones, expressing concern about its aging effect on her appearance. The visual assessment revealed exaggerated subzygomatic arch depression. To address this, the correction was performed using an 18‐gauge, 5 cm N‐scaffold (N‐finders, Korea). Thicker and firmer volume threads, such as the N‐Scaffold (N‐Finders, Korea), are typically employed for subzygomatic arch depression volumization. The patient underwent biweekly follow‐ups for 1‐month post‐thread lift, during which no adverse events or complications were observed. A follow‐up photograph was taken at the 1‐month mark to assess the outcomes (Figure 2).

**FIGURE 2:**
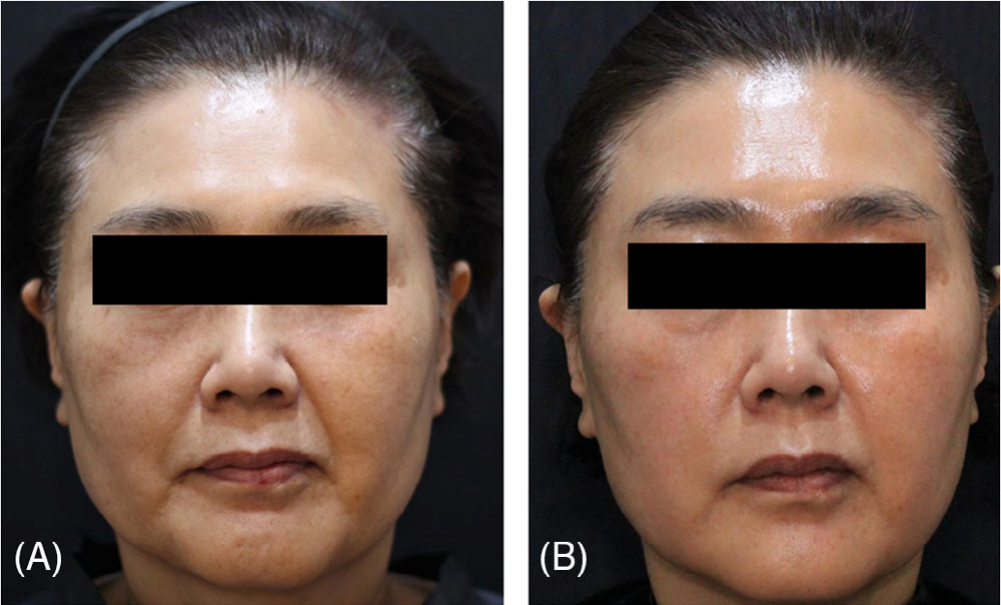
A 45‐year‐old patient visited (B) to correct her subzygomatic arch depression. To address this, a correction was performed using an 18‐gauge, 5 cm N‐scaffold (N‐finders, Korea). The patient's progress was monitored at 2‐week intervals post‐thread insertion, and a follow‐up photo was taken 1 month (B) later to evaluate the outcomes.

The patient expressed satisfaction with the results, both immediately post‐procedure and during the 1‐month follow‐up, highlighting a preference for improved cheek contour and looking more refreshed.

The subzygomatic region comprises the skin, a layer of subcutaneous fat, the SMAS, a sub‐SMAS fatty layer, the parotidomasseteric fascia, and the masseter muscle. Using HA filler in this area poses a risk of vascular damage, especially around the superficial temporal artery and pretragal area.^3^ Hence, given this risk, volumizing thread lifts emerge as a viable alternative worth thorough consideration.^4^, ^5^, ^6^, ^7^, ^8^, ^9^, ^10^ In our clinical practice, we target age‐related subzygomatic volume deficiency and atrophy, alongside subzygomatic depressions, in young Southeast Asian women. This treatment is designed to contour the oval face and diminish subzygomatic prominence through the use of volumizing thread lifts, aligning with the aesthetic preference for an oval face contour often desired by individuals of Southeast Asian descent.^11^, ^12^

Lots ^13^ investigates the anti‐aging effects of polydioxanone (PDO) threads in facelift procedures, involving 10 patients with medium‐grade facial ptosis aged 40−50. PDO threads were implanted, and follow‐ups were conducted over 120 days using ultrasound and photography. Results indicated collagen formation around the threads, skin improvement, and sagging reduction. Physiological and morphological changes in facial aging were discussed alongside PDO threads' effectiveness in collagen formation, tissue repositioning, and vascular improvement. Statistical analysis revealed increased dermal thickness and decreased hypodermic layer post‐thread installation. The study highlights the effectiveness of PDO threads in stimulating collagen formation and repositioning tissue, which can be particularly beneficial for addressing subzygomatic arch depression. The literature extensively explores the role of PDO threads in stimulating collagen formation, with numerous studies supporting this effect.^14^, ^15^, ^16^ For instance, Kim et al.^17^ compared the collagen‐producing effects of powdered PDO with poly‐L‐lactic acid, finding that powdered PDO injections induce collagen formation more effectively in a murine model.

In conclusion, our case presents the effectiveness of addressing subzygomatic arch depression through the application of volumizing PDO threads, showcasing it as an effective and safe technique in facial rejuvenation. The patient's satisfaction with the outcomes highlights the efficacy of this approach in achieving the desired aesthetic improvements. Further investigation is necessary to assess the long‐term safety and effectiveness of PDO thread lift procedures for subzygomatic arch depression.

## CONFLICT OF INTEREST STATEMENT

The authors declared no potential conflicts of interest with respect to the research, authorship, and publication of this article. This study was conducted in compliance with the principles set forth in the Declaration of Helsinki.

## Supporting information

Video S1

## Data Availability

The data that support the findings of this study are available from the corresponding author upon reasonable request.
